# Cardiovascular risk factors are associated with cognitive trajectory in the first year after stroke

**DOI:** 10.1016/j.cccb.2024.100230

**Published:** 2024-06-08

**Authors:** Lena KL Oestreich, Paul Wright, Michael J O'Sullivan

**Affiliations:** a School of Psychology, The University of Queensland, Brisbane, Australia; b Australian Institute for Bioengineering and Nanotechnology, The University of Queensland, Brisbane, Australia; c Centre for Advanced Imaging, The University of Queensland, Brisbane, Australia; d Department of Biomedical Engineering and Imaging Sciences, King's College London, United Kingdom; e Department of Neurology, Royal Brisbane and Women's Hospital, Herston, Australia; f Institute for Molecular Bioscience, University of Queensland, Brisbane, Australia; g National Imaging Facility, University of Queensland, Brisbane, Australia

**Keywords:** Cerebrovascular disease, Stroke, Vascular dementia, Memory, Rehabilitation, Post-stroke cognitive prognosis

## Abstract

**Introduction:**

Stroke often leads to cognitive impairment, but its progression and influencing factors over time remain poorly understood. This study evaluates immediate post-stroke cognitive impacts and investigates the influence of concurrent factors on cognitive evolution over the first year.

**Patients and methods:**

In the STRATEGIC study, 179 patients with first symptomatic ischemic stroke underwent neuropsychological assessments within three months post-stroke, and 141 were re-evaluated at 12 months. Risk factors tested for associations with cognitive outcome included demographic variables, cardiovascular and other medical factors, and lesion characteristics. Cognitive performance was primarily measured via the Montreal Cognitive Assessment (MoCA), with domain-specific assessments for episodic memory (Free and Cued Selective Reminding Task), short-term memory (Digit Span forward), and working memory (Digit Span backward).

**Results:**

At the time of stroke, participants ranged in age from 46 to 89 years (*M* = 70, *SD* = 9.5) and 36.9% were female. Ischemic heart disease predicted cognitive non-improvement between 3 and 12 months. Atrial fibrillation and carotid stenosis were linked to changes in episodic and working memory, respectively. Moreover, female sex and lower education correlated with stagnant global cognition and episodic memory.

**Discussion and conclusion:**

Our findings underscore the important influence of cardiovascular risk factors on cognitive functional recovery after stroke. Interventions targeting these risk factors may improve cognitive prognosis and affect traditional outcome measures such as recurrent vascular events. Future trials should include cognitive measures to fully capture the potential benefits of intensive risk factor intervention.

## Highlights

• We investigated factors that affect cognitive prognosis within one year of stroke.
• Ischemic heart disease was linked to worse cognitive outcome in stroke survivors.
• Atrial fibrillation and carotid stenosis at time of stroke adversely affect memory.
• Cardiovascular risk factors are important modulators of stroke recovery.
• Interventions targeting cardiovascular risk factors may enhance cognitive prognosis.

## 1 Introduction

Cognitive impairment is a common sequel of stroke, with as many as 80% of patients experiencing some level of cognitive decline [1]. Even in the context of mild overall impairment, cognitive deficits can significantly hinder daily living activities and compromise functional independence. Stroke survivors frequently describe difficulties with memory and concentration (43% and 45% respectively), which are more likely to be reported as unmet needs than other long-term complaints such as reduced mobility or speech impairments [2]. While a subset of individuals might see their cognitive functions spontaneously improve [3,4], in a proportion of patients, memory impairments evolve into post-stroke dementia [5]. The early identification of stroke survivors at risk for cognitive decline is crucial for the provision of targeted support and interventions, aiming to enhance their quality of life [6]. However, the prediction of cognitive trajectories remains complicated due to the scarcity of data on influencing factors and the broad range of demographic, neurological, and cardiovascular variables involved. This complexity underscores the need for comprehensive assessments to understand the natural history of cognitive deficits following a stroke, a challenge that has been highlighted in both recent reviews [1,7] and efforts to establish research priorities for stroke recovery [7].

While lesion location is a strong predictor of cognitive impairment in the first 3–6 months after stroke [8], subsequent changes in cognitive performance vary considerably between individuals. This variation underscores the complexity of post-stroke cognitive recovery, which can manifest as improvements, declines, or stability, influenced by the assessment tools used and the timing of follow-up evaluations. Notably, global cognitive function has been observed to decline several years post-stroke, while some studies report cognitive improvements within the first six months. Age and lower levels of education have been consistently linked to cognitive decline [4,9]. However, the influence of other risk factors such as diabetes, atrial fibrillation, hypertension, and smoking presents mixed results across research findings [10]. Moreover, while some studies offer a broad perspective on potential risk factors, they often neglect in-depth characterization of stroke features and lesion attributes [9]. Conversely, studies that describe a relationship with infarct location often fail to affirm these relationships once other factors are considered, suggesting confounding by underlying risk factors [11].

Few studies have concurrently examined demographic, clinical, and other risk factors alongside lesion attributes to discern associations with cognitive prognosis after stroke [12]. Among those that do provide such comprehensive evaluations, follow-up data on long-term cognitive outcomes remains scarce. In this study, our first aim was to compare cognitive profiles between patients who have recently experienced a stroke (within 3 months post-stroke) and healthy controls, as well as to chart the trajectory of cognitive outcomes from 3 to 12 months post-stroke in the patient group. Secondly, we aimed to test the associations between a broad set of potential modifying factors and lesion characteristics, with overall cognitive function, at 3-months post-stroke and cognitive prognosis one year after stroke. Given the frequently reported memory and concentration problems among stroke survivors, we also tested associations of risk factors and lesion characteristics with changes in episodic, short-term, and working memory function from 3 to 12 months post-stroke. We hypothesized that lesion characteristics would be more important early after stroke, but that demographic and cardiovascular risk factors would be relatively more important as predictors of change over time and outcomes at one year.

## 2 Methods

### 2.1 Design

The STRATEGIC study is a prospective observational cohort study designed to follow the cognitive trajectory of stroke patients over the first year post-infarct. This study design was chosen to minimize the potential influence of selection bias given the study objectives. A retrospective design would be vulnerable to this, with patients with better cognitive outcome more likely to be identified in a post-stroke follow up setting. It provides a comprehensive evaluation of cardiovascular risk factors, other medical factors, evaluation of infarct characteristics by MRI and cognitive performance within the first 3-months of stroke. Information on stroke etiology, risk and demographic factors was collected at baseline. A clinical and cognition only follow-up phase provided data on change in global cognitive outcome and domain-specific scores between 3- and 12-months after stroke. Data was collected between January 2015 and April 2019.

### 2.2 Participants

**Fig. 1:**
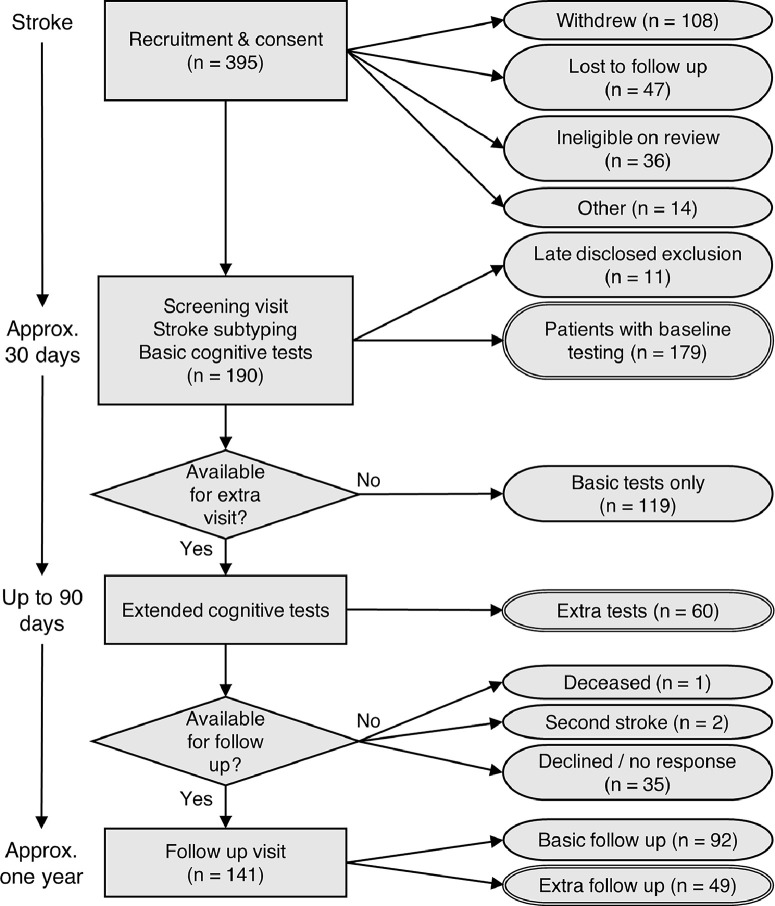
Flowchart of participant inclusion/ exclusion in the study.

A total of 179 patients with first symptomatic ischaemic stroke were recruited within seven days of onset from a single London hyperacute stroke unit (King's College Hospital). All patients admitted to the King's Hyperacute Stroke Unit were screened for eligibility. Screening was performed by a team of research co-ordinators. As for other studies supported by the unit, screening involved evaluation of a checklist of inclusion and exclusion criteria. Review of case notes was performed to complete the checklist so that eligibility was determined by clinical assessments. Inclusion criteria were age over 50 years and acute clinical stroke corroborated by CT or MRI. Exclusion criteria were previous large-artery infarct, dementia, lack of English fluency, active malignancy, major neurological or psychiatric disease, previous moderate to severe head injury and lack of capacity to consent. Patients with impairments of sufficient severity to interfere with cognitive testing, such as severe visual impairment, dyslexia or aphasia were also excluded. Consecutive patients meeting study criteria gave written, informed consent at enrolment. The study was approved by the London and Bromley Research Ethics Committee (ref: 13-LO-1745) and the study is registered with ClinicalTrials.gov at https://clinicaltrials.gov/show/NCT03982147. One patient died before the 1-year follow-up and two others were excluded due to recurrent strokes. Thirty-five participants either declined or did not respond to the invitation for follow-up evaluation. Follow-up data were therefore acquired in 141 of the original sample. The flowchart for recruitment and assessment is shown in Fig. 1. Control participants were recruited from the local community by advertisements, placed in the hospital, university and at local events. A smaller sample of control participants was recruited based on lower variability (heterogeneity) of cognitive scores. The controls were assessed at baseline only, to provide baseline comparison data (to identify acute deficits).

### 2.3 Stroke subtyping, risk factors and infarct delineation

During the baseline visit, a comprehensive medical history was obtained through participant interviews as part of the study procedures. Additionally, a thorough review of the patients’ electronic medical records was conducted, and etiological subtypes of stroke verified or updated based on TOAST classification [13]. Atrial fibrillation (AF) was identified when more than 7 min of irregular heart rhythm with morphology consistent with AF were recorded on an ECG, indicating a common risk factor for stroke. Carotid stenosis was determined when Doppler ultrasound or CT angiography revealed greater than 50% narrowing of the carotid vessels, a critical condition leading to increased stroke risk. Ischemic heart disease (IHD), characterized by reduced blood flow to the heart due to narrowed arteries, increases the risk of stroke by promoting atherosclerosis, clot formation, and conditions that impair cerebral circulation. IHD was identified in individuals who either reported a history of acute coronary syndrome, verified by medical records, or had undergone a revascularization procedure to open blocked arteries. This includes those with an unequivocal history of angioplasty or bypass grafts, or records confirming such procedures, indicating severe heart conditions involving reduced blood flow to the heart. Diabetes mellitus, associated with blood vessel damage due to high sugar levels, and statin use, indicative of cholesterol management efforts, were reviewed from medical records. Smoking and family history of cardiovascular disease were assessed via self-report. Hypertension can damage blood vessels over time, thereby increasing the risk of stroke by making arteries more prone to narrowing, blockage, or rupture, which disrupts normal blood flow to the brain. It was identified by asking patients whether they had been diagnosed with or prescribed medication for high blood pressure. Clinical imaging was reviewed, and infarcts were meticulously classified based on their hemisphere and arterial territory involvement. Distinctions were made between cortical areas affected by the anterior, middle, or posterior cerebral arteries, classified into anterior (ACA), middle (MCA), or posterior (PCA) cerebral artery territories, respectively. Non-thalamic lacunar lesions were defined as lesions in white, or deep gray matter with a diameter <15 mm. Severity of white matter disease on clinical images was graded by a consultant neurologist (MJO) using a simplified Fazekas scale [14]. The scale separately grades periventricular white matter (PWM) and deep white matter (DWM) changes from 0 to 3. Grade 0 indicates no lesions, representing a normal finding. For PWM, Grade 1 signifies caps or pencil-thin lining, Grade 2 reflects smooth “halo” lesions, and Grade 3 is characterized by irregular periventricular signal extending into the deep white matter. In the assessment of DWM, Grade 1 indicates punctate foci, Grade 2 shows the beginning confluence of these foci, and Grade 3 represents large confluent areas. Lesion outlines were defined in 152 participants, 85 on CT (slice thickness: average=4.78 mm, range=2–5 mm) and 67 on MRI (slice thickness: average=4.91 mm, range=1.2–7 mm). Clinical images were acquired at a mean of 6 days after stroke onset (range 1–91 days).

### 2.4 Cognitive evaluation

A battery of neuropsychological tests was performed at the baseline visit: the National Adult Reading Test Revised (NART-R) [15] estimates premorbid intellectual function. Significant changes are measured against individual baselines. The Digit Symbol Substitution Test (DSST), a measure of information processing speed and executive function [16], with scores above the 25th percentile considered normal for adults, and changes over 7 points deemed clinically significant. the Trail-making Test [17], which measures visual search speed and flexibility, suggests normal performance when Part A is completed in under 78 s and Part B in less than 273 s, with a 20% increase in time signaling notable decline. Digit Span, an index of short-term and working memory [16], considers a decrease by 2 or more points from average scores as clinically meaningful. The Free and Cued Selective Reminding Task (FCSRT), a measure of episodic memory [18] and Letter and Category Fluency, testing semantic memory and retrieval [19], use age-adjusted norms for normalcy, viewing a 15% drop as significant. Process Dissociation Procedure [20] and Face Recognition [21] assessing verbal and visuo-spatial learning respectively, mark deviations from normative scores as impairment, with the Corsi Block Test [22] noting declines in visuo-spatial working memory against established norms. The Stroop Test [23], measuring response inhibition and the Iowa Gambling Task [24] to assess decision making, consider performance well below established norms as indicative of clinically meaningful. The MoCA, a general cognitive ability test, labels scores below 26 as cognitive impairment, with a 2-point drop being clinically significant.

At the 12-month follow-up visit, a full medical history was taken. Any new medical events were noted and patients with a history of recurrent stroke were excluded. The MoCA, FCSRT and Digit Span tests were repeated, with a different, counter-balanced, version of the test from the baseline visit.

### 2.5 Analysis and statistics

Statistical analyses were performed using SPSS version 25 (IBM Corp. Armonk, NY). Performance on neuropsychiatric assessments was compared between stroke patients and healthy controls using analyses of variance (ANOVAs). Shapiro-Wilk (preferred for small to medium sample sizes of *n* < 50) and Kolmogorov-Smirnov Tests (suited for larger sample sizes) were used to test normality of test score distributions. Whenever the normality assumption was violated in at least one group, Mann-Whitney U test was used instead of ANOVA. Difference scores on the MoCA, FCSRT and Digit Span were calculated between the 3-month baseline assessment and 12-month follow-up. Participants were classified into groups of patients with cognitive *improvement* (follow-up score>baseline score) or *stable/declining* cognitive performance (follow-up score≤baseline score). In order to assess whether clinical variables, risk factors and/or lesion characteristics taken at baseline could predict cognitive outcome at 12-months, an ordinal logistic regression analysis was performed with the dependent variable *cognitive outcome* (improvement vs stable/decline). Cognitive improvement was set as the reference category to determine factors the lead to a poorer cognitive outcome 1-year after stroke. The independent variables were *atrial fibrillation* (yes/no); *carotid stenosis* (yes/no); *ischemic heart disease* (yes/no); *diabetes mellitus* (yes/no); *hypertension* (yes/no); *statins* (yes/no); *smoking current* (yes/no); *family history of cardiovascular disease* (yes/no); *Fazekas* (0/1/2/3); *lesion hemisphere* (left/right); and *lesion classification - simplified* (non-thalamic lacunar/thalamic/MCA/ACA/PCA). Age, sex, handedness, years of education, NART-R score, baseline cognitive score and days between stroke and cognitive testing were added as covariates into the models. Variance inflation factors (VIF) were assessed to test for multicollinearity between the variables in the regression models. Logistic regression analyses are Bonferroni-corrected for five comparisons.

## 3 Results

### 3.1 Demographics, risk factors and subtypes of stroke

There were 94 right and 85 left hemisphere infarcts. Baseline cognitive evaluation took place at a median 43.5 days after stroke (range 22–109 days). Follow-up evaluation took place at a median of 384 days after stroke (range 355–657 days). The patients with (*n* = 141) and without (*n* = 38) follow-up assessments at 12-months were compared on demographics, cardiovascular risk factors and stroke characteristics (see Table 1), with the only significant difference being that a smaller proportion of smokers was in the group of patients who returned for follow-up assessment.

**Table 1:** Participant demographics, infarct locations and risk factors.

Factor		Baseline and Follow-up	Baseline Only	Statistic	p
n		141	38		NS
Age, mean (SD)		70.0 (9.5)	69.5 (9.0)	t(177) = 0.3	NS
Education, mean (SD)		13.4 (3.7)	12.3 (3.3)	t(170) = 1.8	NS
Female, n (%)		52 (36.9)	8 (21.1)	χ^2^(1,*N* = 179) = 3.4	NS
Handedness	Right	132	37	χ^2^(1,*N* = 179) = 0.8	NS
	Left	9	1		
Ethnicity	Caucasian	122	29	χ^2^(4,*N* = 179) = 6.3	NS
	Non Caucasian	19	9		
Vascular territory	Left ACA	2	0	χ^2^(6,*N* = 179) = 6.4	NS
	Left MCA	39	8		
	Right MCA	45	13		
	Left PCA	7	2		
	Right PCA	10	6		
	Left lacunar	19	2		
	Right lacunar	10	4		
	Left thalamic	4	2		
	Right thalamic	5	1		
Atrial fibrillation	No	104	29	χ^2^(1,*N* = 179) = 0.1	NS
	Yes	37	9		
Carotid stenosis (>50%)	No	125	32	χ^2^(1,*N* = 179) = 0.6	NS
	Yes	16	6		
Diabetes mellitus	No	112	29	χ^2^(1,*N* = 179) = 0.2	NS
	Yes	29	9		
Hypertension	No	50	10	χ^2^(1,*N* = 179) = 1.1	NS
	Yes	91	28		
Ischaemic heart disease	No	110	29	χ^2^(3,*N* = 179) = 0.1	NS
	Yes	31	9		
Smoking at stroke	Never	74	11	**χ^2^(3,*N* = 179) = 10.8**	**0.01**
	Quit	43	14		
	Current	24	13		
Fazekas score	0	12	4	χ^2^(3,*N* = 179) = 0.5	NS
	1	57	14		
	2	46	14		
	3	26	6		

*Note.* ACA: anterior cerebral artery. MCA: middle cerebral artery. PCA: posterior cerebral artery.

Patients and healthy controls were matched on age, handedness and years of education, but differed significantly on sex (χ^2^(1)=16.7, *p* < 0.001), with a larger proportion of females in the healthy control group (72% female) than the group of stroke patients (34% female). Natal sex was therefore added as a covariate to all analyses including healthy controls.

### 3.2 Comparison of neuropsychological test scores between stroke patients 3-months post stroke and healthy controls

Comparisons of neuropsychological test results between healthy controls and patients are presented in Table 2. Patients exhibited significantly lower scores than healthy controls on FCSRT delayed and total recall, Trails A, Trails B, DSST, Digit Span Forward, and Letter Fluency. These findings indicate that patients exhibited impairment in attention, memory, and executive functioning. Specifically, impairments were pronounced in verbal episodic memory, visual attention and task switching, processing speed and sustained attention, as well as retrieval from long-term memory. Relative to health controls, patients were relatively unimpaired on tasks involving short-term memory, visuo-spatial and verbal working memory, semantic memory, learning, inhibition, and decision making.

**Table 2:** Neuropsychological scores by group and group comparisons.

Test	Healthy controls		Stroke patients		Group comparison
	n	mean (SD)	n	mean (SD)	
NART-IQ	32	118.19 (8.99)	165	110.52 (10.92)	*U* = 3820.5, *p* < **0.001**
FCSRT delayed recall	32	34.63 (9.08)	177	27.1 (7.83)	*U* = 4204.5, *p* < **0.001**
FCSRT free/ total recall	32	0.74 (0.17)	177	0.61 (0.17)	*U* = 4166.5, *p* < **0.001**
Trails A (s)	32	41.03 (14.89)	169	57.79 (30.01)	*U* = 1732, *p* = **0.001**
Trails B (s)	32	100.41 (53.44)	169	157.94 (87.68)	*U* = 1633, *p* < **0.001**
DSST	32	48.22 (15.04)	167	35.35 (11.95)	*U* = 3914, *p* < **0.001**
Digit span forward	29	6.72 (1.07)	174	6.18 (1.1)	*U* = 31,940.5, *p* = **0.017**
Digit span backward	28	4.93 (1.19)	174	4.45 (1.19)	*U* = 2953.5, *p* = 0.062
Letter fluency	30	16.46 (4.61)	52	13.34 (4.76)	*F*(1,79) = 10.88, *p* = **0.026**
Category fluency	26	19.94 (6.64)	52	16.46 (4.64)	*F*(1,75) = 8.46, *p* = 0.086
Process dissociation - recollection	32	0.45 (0.28)	49	0.37 (0.23)	*F*(1,78) = 0.03, *p* = 0.99
Process dissociation - familiarity	32	0.4 (0.19)	49	0.29 (0.19)	*F*(1,78) = 2.97, *p* = 0.99
Face recognition - remember	32	13.47 (7.71)	57	20.51 (10.86)	*F*(1,86) = 7.8, *p* = 0.99
Face recognition - familiarity	32	10.56 (5.55)	57	7.75 (6.08)	*F*(1,86) = 5.5, *p* = 0.378
Corsi span	30	4.87 (0.78)	52	4.58 (0.96)	*U* = 945.5, *p* = 0.082
Stroop – naming incongruent (ms)	30	1673.63 (571.6)	36	1843.69 (822.9)	*U* = 406.5, *p* = 0.07
Stroop – reading incongruent (ms)	30	1274.3 (307.51)	36	1435.72 (607.46)	*U* = 411.5, *p* = 0.096
Iowa gambling task	27	1806.48 (916.9)	24	1581.04 (652.36)	*F*(1,48) = 1.08, *p* = 0.99

*Note. n* = sample size; SD = standard deviation; *U* = Mann-Whitney U Test, used in case of deviation from normal distribution in at least one group. NART-IQ = national adult reading test – intelligence quotient; FCSRT = free and cured selective reminding task; DSST = digit symbol substitution task; *s* = seconds; ms = milliseconds; Bonferroni corrected for 18 comparisons.

### 3.3 Cognitive trajectories from 3- to 12- months post-stroke

**Fig. 2:**
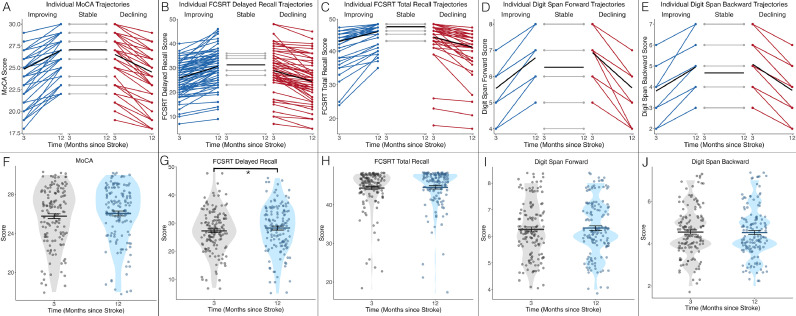
Individual trajectories (A-E) and longitudinal comparisons (F-J) of Montreal Cognitive Assessment (MoCA), Free and Cued Selective Reminding Task (FCSRT) and Digit Span scores.

Patients’ baseline MoCA scores ranged from 18 to 30 out of 30 (*M* = 25.77, *SD* = 2.99). MoCA scores at follow-up also ranged from 18 to 30 (*M* = 26.04, *SD* = 2.96). While the difference between mean baseline and follow-up scores was not significant (*F*(1140) = 2.17, *p* = 0.143), both improvement and deterioration were observed at follow-up. Scores improved in 69 (49%) and remained stable or declined in 72 (51%) patients. Baseline FCSRT scores ranged from 7 to 48 out of 48 for delayed recall (*M* = 27.26, *SD* = 7.74) and 18 to 48 for total recall (*M* = 44.36, *SD* = 5.24). Follow-up scores ranged from 5 to 46 for delayed (*M* = 28.26, *SD* = 8.46) and 17 to 48 for total recall (*M* = 44.54, *SD* = 8.46). Mean FCSRT scores significantly increased for delayed recall (*F*(1140) = 4.6, *p* = 0.034), but not for total recall (*F*(1139) = 0.38, *p* = 0.539). Both improvement and decline in episodic memory were observed at follow-up, whereby scores on delayed recall improved in 79 (56%) and remained stable or declined in 62 (44%) participants. Scores on total recall improved in 59 (42.1%) and remained stable/declined in 81 (57.9%) participants. Digit Span forward scores ranged from 4 to 8 out of 8 at baseline (*M* = 6.26, *SD*=1.08) and 4 to 8 at follow-up (*M* = 6.31, *SD*=1.03). Digit Span backward scores ranged from 2 to 7 out of 7 at baseline (*M* = 4.55, *SD* = 1.19) and 2 to 7 at follow-up (*M* = 4.52, *SD* = 1.16). Mean scores on the Digit Span did not change significantly for the forward (*F*(1136) = 0.98, *p* = 0.324) or the backward (*F*(1136) = 0.14, *p* = 0.712) conditions. We observed improvement on short-term memory as measured by the Digit Span forward in 35 (25.5%) and stable or declining short-term memory in 102 (74.5%) participants. Improved working memory, measured by the Digit Span backward, was observed in 36 (26.3%) and stable/declining working memory in 101 (73.7%) participants. Individual trajectories and longitudinal comparisons are displayed in Fig. 2.

### 3.4 Associations of risk factors and lesion characteristics with overall cognitive change from 3- to 12- months post-stroke

VIFs across regression analyses ranged from 1.06 to 2.82 for both covariates and independent variables in all models, indicating no concerns with multicollinearity.

A logistic regression with change in MoCA score produced a model that was significant in predicting cognitive outcome (χ^2^(23) = 51.49, *p* = 0.003, Bonferroni corrected). The cardiovascular risk factor *Ischemic heart disease* (χ^2^(1) = 8.26, *p* = 0.004), emerged as a significant independent predictor of cognitive prognosis, whereby patients with a history of ischemic heart disease were 8 times more likely to have a decline in cognitive outcome relative to those free of ischemic heart disease. Moreover, the demographic variables *natal sex* (χ^2^(1) = 5.73, *p* = 0.017) and *years of education* (χ^2^(1) = 7.45, *p* = 0.006) emerged as significant independent predictors, such that females were almost 6 times more likely to have a poor cognitive outcome relative to males. Moreover, fewer years of education was associated with a lack of improvement in MoCA score. The final model correctly classified 76.1% of patients into groups of patients with stable/declining versus improving cognitive trajectories. Odds ratios and p-values of the parameter estimates are presented in Table 3.

**Table 3:** Odds of stable/worsening vs improved cognitive outcome.

Factor	Odds ratio	p-value
Age	0.98	0.49
Sex (female vs male)	**3.08**	**0.017**
Handedness (right vs left)	1.29	0.82
Years of education	**0.78**	**0.006**
NART-R (verbal IQ)	1.1	0.01
MoCA baseline score	**0.61**	**< 0.001**
Days since stroke	1.01	0.44
Lesion hemisphere (left vs right)	1.77	0.24
Vascular territory (reference category thalamic)		
ACA	0	0.99
MCA	0	0.99
PCA	0	0.99
Non-thalamic lacunar	0	0.99
Fazekas (reference category 0)		
1	1.15	0.89
2	2.12	0.4
3	2.06	0.42
Atrial fibrillation (no vs yes)	1.93	0.23
Smoking - current (no vs yes)	1.44	0.44
Family history (no vs yes)	2.81	0.09
IHD (no vs yes)	**6.9**	**0.004**
Diabetes mellitus (no vs yes)	1.23	0.59
Carotid stenosis (no vs yes)	1.42	0.63
Hypertension (no vs yes)	1.28	0.64
Statins (no vs yes)	1.89	0.46

*Note.* NART-R: national adult reading test - revised; ACA: anterior cerebral artery, MCA: middle cerebral artery, PCA: posterior cerebral artery.

### 3.5 Associations of risk factors and lesion characteristics with episodic memory change from 3- to 12- months post-stroke

The logistic regressions with FCSRT delayed and total recall as outcome variables did not produce models that remained significant after Bonferroni correction. However, the cardiovascular risk factor *atrial fibrillation* (χ^2^(1) = 3.11, *p* = 0.034) emerged as a significant independent predictor of change in delayed recall, whereby patients with atrial fibrillation were 3 times more likely to have stable or declining episodic memory over follow-up relative to those free of atrial fibrillation. While none of the covariates were independent predictors of delayed recall, *natal sex* (χ^2^(1) = 4.36, *p* = 0.039) and *years of education* (χ^2^(1) = 3.89, *p* = 0.044) were significant independent predictors of total recall, whereby women were over 4 times more likely than men to demonstrate a decline in episodic memory. Furthermore, fewer years of education were predictive of worsening episodic memory. The final models correctly classified 71.6% (FCSRT delayed recall) and 68.7% (FCSRT total recall) of patients into groups of those with stable/declining versus improving cognitive trajectories. Odds ratios and p-values of the parameter estimates are presented in Table 4, Table 5.

**Table 4:** Odds of stable/worsening vs improved episodic memory outcome (delayed recall).

Factor	Odds ratio	p-value
Age	0.98	0.39
Sex (female vs male)	1	1
Handedness (right vs left)	4.47	0.13
Years of education	0.86	0.07
NART (verbal IQ)	1.04	0.14
FCSRT delayed recall baseline score	0.94	0.044
Days since stroke	1	0.78
Lesion hemisphere (left vs right)	1.53	0.37
Vascular territory (reference category thalamic)		
ACA	0	0.99
MCA	0	0.99
PCA	0	0.99
Non-thalamic lacunar	0	0.99
Fazekas (reference category 0)		
1	0.78	0.75
2	1.38	0.62
3	1.02	0.99
Atrial fibrillation (no vs yes)	**3.11**	**0.034**
Smoking - current (no vs yes)	1.02	0.97
Family history (no vs yes)	1.42	0.54
IHD (no vs yes)	0.54	0.24
Diabetes mellitus (no vs yes)	0.55	0.3
Carotid stenosis (no vs yes)	1.17	0.82
Hypertension (no vs yes)	1.5	0.39
Statins (no vs yes)	2.2	0.36

*Note.* NART-R: national adult reading test - revised; ACA: anterior cerebral artery, MCA: middle cerebral artery, PCA: posterior cerebral artery.

**Table 5:** Odds of stable/worsening vs improved episodic memory outcome (total recall).

Factor	Odds ratio	p-value
Age	0.99	0.74
Sex (female vs male)	**4.31**	**0.039**
Handedness (right vs left)	3.55	0.19
Years of education	**0.85**	**0.044**
NART-R (verbal IQ)	1.03	0.3
FCSRT total recall baseline score	1.02	0.74
Days since stroke	1	1
Lesion hemisphere (left vs right)	1.25	0.63
Vascular territory (reference category thalamic)		
ACA	0	0.99
MCA	0	0.99
PCA	0	0.99
Non-thalamic lacunar	0	0.99
Fazekas (reference category 0)		
1	1.07	0.93
2	1.13	0.85
3	1.21	0.8
Atrial fibrillation (no vs yes)	1.68	0.11
Smoking - current (no vs yes)	0.96	0.93
Family history (no vs yes)	1.17	0.77
IHD (no vs yes)	1.69	0.31
Diabetes mellitus (no vs yes)	1.17	0.32
Carotid stenosis (no vs yes)	1.45	0.84
Hypertension (no vs yes)	1.73	0.31
Statins (no vs yes)	0.28	0.51

*Note.* NART-R: national adult reading test - revised; ACA: anterior cerebral artery, MCA: middle cerebral artery, PCA: posterior cerebral artery.

### 3.6 Associations of risk factors and lesion characteristics with short-term and working memory change from 3- to 12- months post-stroke

The logistic regression with Digit Span forward as outcome variable was not significant overall and none of the covariates or predictors individually significantly predicted short-term memory. The logistic regression with Digit Span backward as outcome variable significantly predicted working memory (χ^2^(23) = 45.32, *p* = 0.032, Bonferroni corrected). The cardiovascular risk factor *carotid stenosis* (χ^2^(1) = 9.23, *p* = 0.017) emerged as a significant independent predictor of working memory, whereby patients with carotid stenosis were over 9 times more likely to exhibit deteriorating working memory relative to those free of carotid stenosis. The final models correctly classified 82.3% (Digit Span forward) and 78.5% (Digit Span backward) of stroke participants into groups of patients with stable/declining versus improving cognitive trajectories. Odds ratios and p-values of the parameter estimates are presented in Table 6, Table 7.

**Table 6:** Odds of stable/worsening vs improved short term memory.

Factor	Odds ratio	p-value
Age	1.02	0.57
Sex (female vs male)	1.27	0.74
Handedness (right vs left)	1	1
Years of education	1.16	0.17
NART-R (verbal IQ)	0.96	0.19
Digit Span Forward baseline score	**3.4**	**0.002**
Days since stroke	1	0.92
Lesion hemisphere (left vs right)	0.43	0.21
Vascular territory (reference category thalamic)		
ACA	0	0.99
MCA	0	0.99
PCA	0	0.99
Non-thalamic lacunar	0	0.99
Fazekas (reference category 0)		
1	2.19	0.63
2	4.78	0.24
3	3.69	0.33
Atrial fibrillation (no vs yes)	1.02	0.98
Smoking - current (no vs yes)	1.52	0.51
Family history (no vs yes)	4	0.09
IHD (no vs yes)	2.22	0.39
Diabetes mellitus (no vs yes)	1.93	0.49
Carotid stenosis (no vs yes)	2.17	0.46
Hypertension (no vs yes)	1.94	0.31
Statins (no vs yes)	2.62	0.34

*Note.* NART-R: national adult reading test - revised; ACA: anterior cerebral artery, MCA: middle cerebral artery, PCA: posterior cerebral artery.

**Table 7:** Odds of stable/worsening vs improved working memory.

Factor	Odds ratio	p-value
Age	0.99	0.86
Sex (female vs male)	0.87	0.83
Handedness (right vs left)	0.97	0.98
Years of education	1.07	0.49
NART-R (verbal IQ)	0.98	0.18
Digit Span Backward baseline score	**2.55**	**0.002**
Days since stroke	1.03	0.41
Lesion hemisphere (left vs right)	1.23	0.72
Vascular territory (reference category thalamic)		
ACA	0	0.99
MCA	0	0.99
PCA	0	0.99
Non-thalamic lacunar	0	0.99
Fazekas (reference category 0)		
1	0.23	0.06
2	0.52	0.38
3	0.24	0.19
Atrial fibrillation (no vs yes)	1.74	0.45
Smoking - current (no vs yes)	1.74	0.31
Family history (no vs yes)	0.24	0.09
IHD (no vs yes)	2.3	0.22
Diabetes mellitus (no vs yes)	1.78	0.25
Carotid stenosis (no vs yes)	**9.23**	**0.017**
Hypertension (no vs yes)	1.73	0.35
Statins (no vs yes)	0.85	0.88

*Note.* NART-R: national adult reading test - revised; ACA: anterior cerebral artery, MCA: middle cerebral artery, PCA: posterior cerebral artery.

## 4 Discussion

In this study, we investigated cognitive trajectories of patients following a stroke. Our results showed that impairment was common in the subacute phase and occurred across several cognitive domains. While impairment was consistent in some cognitive sub-domains, others remained relatively intact. In the period between initial and 12-month follow-up assessment, there was evidence of both improvement and deterioration of cognitive scores for individual participants. Cardiovascular risk factors identified within the first 3-months post-stroke stood out as reliable predictors of cognitive outcomes one year after stroke. A history of ischaemic heart disease emerged as a significant independent predictor of global cognitive prognosis. Analysis of individual cognitive domains suggested distinct cardiovascular risk factors for specific cognitive outcomes: while ischemic heart disease was associated with an overall cognitive decline, atrial fibrillation and carotid stenosis were linked to episodic and working memory, respectively. Notably, patients presenting these risk factors typically exhibited a poorer cognitive prognosis. In contrast, those devoid of these risk factors often showed signs of cognitive improvement. Beyond cardiovascular considerations, demographic factors such as natal sex and educational attainment also played a pivotal role in cognitive outcomes. Specifically, being female and having fewer years of formal education were risk factors for overall cognitive, and episodic memory decline.

Multiple cognitive domains can be affected by stroke [25], with the overall pattern of involvement varying from person to person. The finding of deficits in areas like verbal episodic memory, visual attention, and task switching highlights the profound and varied cognitive impacts stroke can have, a variability that may be missed in the traditional focus on motor deficits in the clinical and rehabilitation setting. While we observed impairment across a range of tests, we also identified sub-domains that were relatively unimpaired. Specifically, difficulties retrieving information from long-term memory and episodic memory impairments were common among ischemic stroke patients, but short-term and working memory were largely preserved. Similarly, patients performed poorly on executive function tasks involving task switching but performed close to normal on tasks requiring inhibition and decision making. Performance on tasks assessing sub-domains of attention and processing speed were on average impaired across most stroke patients. Cognitive processes are strongly depended on one another and rarely function in isolation. It is therefore possible that difficulties concentrating, and prolonged periods of time needed to process new information, exert spill-over effects onto memory and executive function. However, at the same time, these co-dependencies between cognitive domains may offer an opportunity for plasticity, whereby preserved cognitive functions may compensate for the deterioration of other cognitive domains [26].

The distinct associations between cardiovascular risk factors and cognitive outcomes underscore the intricate relationship between vascular and brain health. Ischemic heart disease emerged as a predictor for a decline in overall cognition, possibly due to its systemic implications on cerebral blood flow and microvascular alteration [27]. This aligns with the ARCOS-IV study which identified associations between coronary heart disease and cognitive outcomes, emphasizing the heart-brain axis's pivotal role in brain health (Mahon et al., 2017). Interestingly, carotid stenosis, which directly impacts cerebral perfusion, was linked to working memory deficits. Working memory depends on an interconnected bilateral network and this result might reflect the fact that diminished perfusion in either hemisphere can disrupt this network. In contrast, the association of atrial fibrillation with episodic memory could be attributed to unidentified embolic events affecting episodic memory circuits that are strongly lateralised to the left hemisphere for verbal memory. Underpinning these associations may be mechanisms such as compromised left ventricular function, which might disturb cerebral circulation [28]. An alternative explanation is that ischemic heart disease, by limiting exercise tolerance, might impede rehabilitation engagement and the anticipated positive effects of exercise on neural plasticity. Collectively, these findings highlight the need for tailored vascular interventions based on specific cognitive vulnerabilities in stroke survivors to potentially mitigate further cognitive decline.

Our study discerned several associations between demographic variables and specific cognitive outcomes. Notably, natal sex and years of education emerged as significant predictors for overall cognitive prognosis and episodic memory performance. Specifically, women were over four times more likely than men to experience a deterioration in both overall cognition and episodic memory. This disparity between the sexes is consistent with previous findings [29], which also reported a pronounced vulnerability in women regarding cognitive decline following a stroke. The reasons behind this disparity remain unknown, but factors such as hormonal differences, genetic predispositions, and varying neural structures might play a role. Number of years spent in formal education emerged as another crucial predictor of post-stroke cognitive outcomes. This observation aligns with a study [30] that highlighted the protective effects of education on cognitive health. Education, in this context, can be viewed as a form of cognitive reserve—a buffer that allows individuals to maintain cognitive function despite potential brain damage. A higher educational background might offer a form of resilience, allowing the brain to tap into alternative neural pathways or strategies to maintain cognitive function.

Hemispheric distinction did not emerge as a significant predictor of cognitive outcomes. This is a noteworthy observation, especially when placed in the context of prior research, which has often underscored differences between left and right hemisphere strokes as instrumental in determining cognitive trajectories post-stroke [11]. Historically, the left hemisphere has been primarily associated with linguistic abilities, while the right has been linked to visuospatial functions, leading to assumptions regarding the specificity of cognitive deficits based on lesion location [31]. Yet, our findings align with a growing body of evidence suggesting that cognitive outcomes following stroke are multifactorial, influenced not solely by hemisphere but by an interplay of factors such as lesion size, neural plasticity, and underlying neurocognitive reserves [32]. As such, our results challenge the conventional hemisphere-based paradigm and propose that, at least for certain cohorts, the hemisphere of injury may not always be the predominant predictor of post-stroke cognitive prognosis. This shift in understanding underscores the need for individualized cognitive assessments and interventions that account for a multitude of influencing factors and indicates that broad generalizations about rehabilitation potential based on hemisphere of stroke should be avoided.

Unlike previous studies, our research highlights associations with cognitive performance early after stroke, as well as the evolution of cognitive performance over time. This led to the discovery that the factors influencing cross-sectional performance and longitudinal change are distinct. This distinction is pivotal given the dynamics of post-stroke neurological recovery. Such recovery, characterized by angiogenesis, synaptogenesis, axonal sprouting, improvement from diaschisis, and cortical reorganization, is most pronounced approximately one to three months post-infarction [33]. Furthermore, while a proportion of stroke survivors demonstrate neurological recovery signs for up to a year, the magnitude of this recovery diminishes over the first 3–6 months post-stroke [34]. Beyond this period, functional recovery, characterized by relearning lost or compromised capabilities and reclaiming independence, takes precedence. The contrasting significance of different risk factors on cognitive prognosis at the 3- and 12-month post-stroke intervals likely mirrors these unique correlations with neurological versus functional recovery. Consequently, our findings underscore the potential for enhancing the accuracy of long-term post-stroke cognitive prognosis. This can be achieved by holistically considering factors tied to both neurological and functional recovery.

The strengths of the present study include a design, which allowed risk and demographic factors to be considered in parallel with infarct characteristics, leading to an understanding of independent associations unlikely to arise from confounding. The use of comprehensive neuropsychological testing, made it possible to contrast the pattern of associations for general cognitive function and specific domains, providing evidence that some associations are domain specific. There are also weaknesses. The recruitment of patients with mild to moderate stroke without overt aphasia places limits on the generalisability of the findings. Lack of insight into pre-stroke cognitive function is a limitation common to many hospital-based cohort studies. Although symptomatic recurrent stroke was excluded by review of clinical histories and medical records, new asymptomatic infarcts were not excluded by serial MRI.

This study underscores the critical interplay between cardiovascular health and cognitive recovery post-stroke, highlighting the potential of targeted cardiovascular interventions to enhance cognitive outcomes. Our findings reveal that specific cardiovascular risk factors, such as ischemic heart disease, atrial fibrillation, and carotid stenosis, significantly predict cognitive decline in stroke survivors. These associations suggest that managing these conditions could be pivotal in mitigating cognitive deterioration and improving overall recovery. Furthermore, the influence of demographic factors, particularly natal sex and educational attainment, on cognitive trajectories post-stroke, emphasizes the need for personalized rehabilitation strategies that consider these variables. Given the substantial proportion of stroke survivors experiencing moderate disability despite advances in acute stroke care, our study advocates for the integration of cognitive assessments into standard post-stroke care protocols. By doing so, healthcare providers can identify at-risk individuals early, enabling the implementation of comprehensive, multidisciplinary interventions that address both the neurological and functional aspects of recovery. Ultimately, incorporating strategies aimed at optimizing cardiovascular health within stroke rehabilitation programs could significantly improve cognitive outcomes, enhancing the quality of life for stroke survivors.

## CRediT authorship contribution statement

**Lena KL Oestreich:** Writing – original draft, Visualization, Investigation, Formal analysis. **Paul Wright:** Project administration, Formal analysis, Data curation. **Michael J O'Sullivan:** Supervision, Resources, Funding acquisition, Conceptualization.

## Declaration of competing interest

The authors have no conflicts of interest to declare.

## Appendix Supplementary materials

Image, application 1
