# Supplementary material for: Cardiovascular risk factors are associated with cognitive trajectory in the first year after stroke
Source: Cereb Circ Cogn Behav. 2024 Jun 8;7:100230. doi: 10.1016/j.cccb.2024.100230 (PMC11231527; doi:10.1016/j.cccb.2024.100230)
Supplement: Supplementary file 1 [file mmc1.docx]

Supplementary Table 1. Odds of stable/worsening vs improved cognitive outcome

| **Factor** | **Odds ratio** | **p-value** |
| --- | --- | --- |
| Age | 0.99 | 0.6 |
| Sex (female vs male) | **3.08** | **0.04** |
| Handedness (right vs left) | 01.29 | 0.8 |
| Years of education | **0.79** | **0.007** |
| NART-R (verbal IQ) | 1.1 | 0.1 |
| MoCA baseline score | **0.61** | **< 0.001** |
| Lesion hemisphere (left vs right) | 1.4 | 0.46 |
| Vascular territory (reference category thalamic)  ACA  MCA  PCA  Non-thalamic lacunar | 0  0  0  0 | 0.99  0.99  0.99  0.99 |
| Fazekas (reference category 0)  1  2  3 | 1.33  1.93  1.92 | 0.74  0.41  0.41 |
| Atrial fibrillation (no vs yes) | 1.86 | 0.24 |
| Smoking - current (no vs yes) | 1.41 | 0.45 |
| Family history (no vs yes) | 2.81 | 0.07 |
| IHD (no vs yes) | **6.09** | **0.005** |
| Diabetes mellitus (no vs yes) | 1.39 | 0.58 |
| Carotid stenosis (no vs yes) | 1.64 | 0.47 |
| Hypertension (no vs yes) | 0.87 | 0.78 |
| Statins (no vs yes) | 1.69 | 0.54 |

*Note.* NART-R: national adult reading test - revised; ACA: anterior cerebral artery, MCA: middle cerebral artery, PCA: posterior cerebral artery.

Supplementary Table 2. Odds of stable/worsening vs improved episodic memory outcome (delayed recall)

| **Factor** | **Odds ratio** | **p-value** |
| --- | --- | --- |
| Age | 0.98 | 0.42 |
| Sex (female vs male) | 1.16 | 0.76 |
| Handedness (right vs left) | 4.62 | 0.11 |
| Years of education | 0.9 | 0.17 |
| NART (verbal IQ) | 1.0  1.03 | 0.34 |
| FCSRT delayed recall baseline score | 0.917 | 0.009 |
| Lesion hemisphere (left vs right) | 1.31 | 0.56 |
| Vascular territory (reference category thalamic)  ACA  MCA  PCA  Non-thalamic lacunar | 0  0  0  0 | 0.99  0.99  0.99  0.99 |
| Fazekas (reference category 0)  1  2  3 | 0.77  2.74  1.03 | 0.74  0.16  0.97 |
| Atrial fibrillation (no vs yes) | **3.16** | **0.028** |
| Smoking - current (no vs yes) | 0.7 | 0.47 |
| Family history (no vs yes) | 1.96 | 0.22 |
| IHD (no vs yes) | 0.74 | 0.55 |
| Diabetes mellitus (no vs yes) | 0.51 | 0.23 |
| Carotid stenosis (no vs yes) | 1.34 | 0.65 |
| Hypertension (no vs yes) | 1.57 | 0.32 |
| Statins (no vs yes) | 1.81 | 0.48 |

*Note.* NART-R: national adult reading test - revised; ACA: anterior cerebral artery, MCA: middle cerebral artery, PCA: posterior cerebral artery.

Supplementary Table 3. Odds of stable/worsening vs improved episodic memory outcome (total recall)

| **Factor** | **Odds ratio** | **p-value** |
| --- | --- | --- |
| Age | 0.97 | 0.14 |
| Sex (female vs male) | **4.39** | **0.036** |
| Handedness (right vs left) | 0.86 | 0.85 |
| Years of education | **0.85** | **0.033** |
| NART-R (verbal IQ) | 1.03 | 0.33 |
| FCSRT total recall baseline score | 0.91 | 0.058 |
| Lesion hemisphere (left vs right) | 0.41 | 0.39 |
| Vascular territory (reference category thalamic)  ACA  MCA  PCA  Non-thalamic lacunar | 1.06  2.53  1.32  5.14 | 0.97  0.58  0.86  0.36 |
| Fazekas (reference category 0)  1  2  3 | 0.58  0.52  0.41 | 0.4  0.31  0.29 |
| Atrial fibrillation (no vs yes) | 1.68 | 0.11 |
| Smoking - current (no vs yes) | 0.72 | 0.95 |
| Family history (no vs yes) | 0.69 | 0.96 |
| IHD (no vs yes) | 2.2 | 0.15 |
| Diabetes mellitus (no vs yes) | 1.17 | 0.82 |
| Carotid stenosis (no vs yes) | 2.83 | 0.23 |
| Hypertension (no vs yes) | 0.84 | 0.92 |
| Statins (no vs yes) | 0.28 | 0.31 |

*Note.* NART-R: national adult reading test - revised; ACA: anterior cerebral artery, MCA: middle cerebral artery, PCA: posterior cerebral artery.

Supplementary Table 4. Odds of stable/worsening vs improved short term memory

| **Factor** | **Odds ratio** | **p-value** |
| --- | --- | --- |
| Age | 1.04 | 0.31 |
| Sex (female vs male) | 1.31 | 0.66 |
| Handedness (right vs left) | 0.89 | 0.91 |
| Years of education | 1.06 | 0.53 |
| NART-R (verbal IQ) | 1.05 | 0.21 |
| Digit Span Forward baseline score | 0.24 | < 0.001 |
| Lesion hemisphere (left vs right) | 1.73 | 0.35 |
| Vascular territory (reference category thalamic)  ACA  MCA  PCA  Non-thalamic lacunar | 0  0  0  0 | 0.99  0.99  0.99  0.99 |
| Fazekas (reference category 0)  1  2  3 | 0.33  0.29  0.48 | 0.3  0.23  0.45 |
| Atrial fibrillation (no vs yes) | 1.57 | 0.43 |
| Smoking - current (no vs yes) | 1.66 | 0.32 |
| Family history (no vs yes) | 1.68 | 0.42 |
| IHD (no vs yes) | 1.60 | 0.41 |
| Diabetes mellitus (no vs yes) | 2.62 | 0.13 |
| Carotid stenosis (no vs yes) | 1.33 | 0.74 |
| Hypertension (no vs yes) | 1.15 | 0.79 |
| Statins (no vs yes) | 0.26 | 0.1 |

*Note.* NART-R: national adult reading test - revised; ACA: anterior cerebral artery, MCA: middle cerebral artery, PCA: posterior cerebral artery.

Supplementary Table 5. Odds of stable/worsening vs improved working memory

| **Factor** | **Odds ratio** | **p-value** |
| --- | --- | --- |
| Age | 0.99 | 0.85 |
| Sex (female vs male) | 1.44 | 0.55 |
| Handedness (right vs left) | 1.33 | 0.8 |
| Years of education | 0.94 | 0.54 |
| NART-R (verbal IQ) | 1.09 | 0.28 |
| Digit Span Backward baseline score |  | < 0.001 |
| Lesion hemisphere (left vs right) | 0.8 | 0.68 |
| Vascular territory (reference category thalamic)  ACA  MCA  PCA  Non-thalamic lacunar | 0  0  0  0 | 0.99  0.019  0.21  0.025 |
| Fazekas (reference category 0)  1  2  3 | 0.87  0.98  0.12 | 0.74  0.41  0.41 |
| Atrial fibrillation (no vs yes) | 1.73 | 0.42 |
| Smoking - current (no vs yes) | 1.02 | 0.98 |
| Family history (no vs yes) | 0.87 | 0.83 |
| IHD (no vs yes) | 2.4 | 0.271 |
| Diabetes mellitus (no vs yes) | 1.29 | 0.73 |
| Carotid stenosis (no vs yes) | **9.39** | **0.013** |
| Hypertension (no vs yes) | 1.66 | 0.38 |
| Statins (no vs yes) | 0.47 | 0.45 |

*Note.* NART-R: national adult reading test - revised; ACA: anterior cerebral artery, MCA: middle cerebral artery, PCA: posterior cerebral artery.
